# Genotypic Determination of Extended Spectrum β-Lactamases and Carbapenemase Production in Clinical Isolates of *Klebsiella pneumoniae* in Southwest Nigeria

**DOI:** 10.3390/idr15030034

**Published:** 2023-06-20

**Authors:** Gbolabo Odewale, Motunrayo Yemisi Jibola-Shittu, Olusola Ojurongbe, Rita Ayanbolade Olowe, Olugbenga Adekunle Olowe

**Affiliations:** 1 Department of Microbiology, Federal University, Lokoja P.M.B. 1154, Kogi State, Nigeria; odewalegbolabo@gmail.com (G.O.); motunjibolashittu@gmail.com (M.Y.J.-S.); 2 Department of Medical Microbiology and Parasitology, Ladoke Akintola University of Technology, Ogbomoso P.M.B. 4000, Oyo State, Nigeria; oojurongbe@lautech.edu.ng (O.O.); olowerita@gmail.com (R.A.O.); 3 Centre for Emerging and Re-Emerging Infectious Diseases (CERID-LAUTECH), Ladoke Akintola University of Technology, Ogbomoso P.M.B. 4000, Oyo State, Nigeria

**Keywords:** *Klebsiella pneumoniae*, extended spectrum β-lactamase, carbapenemase genes, multi-locus sequencing typing, polymerase chain reaction

## Abstract

Introduction: *Klebsiella pneumoniae* is a major pathogen implicated in healthcare-associated infections. Extended-spectrum β-lactamase (ESBL) and carbapenemase-producing *K. pneumoniae* isolates are a public health concern. This study investigated the existence of some ESBL and carbapenemase genes among clinical isolates of *K. pneumoniae* in Southwest Nigeria and additionally determined their circulating clones. Materials and Methods: Various clinical samples from 420 patients from seven tertiary hospitals within Southwestern Nigeria were processed between February 2018 and July 2019. These samples were cultured on blood agar and MacConkey agar, and the isolated bacteria were identified by Microbact GNB 12E. All *K. pneumoniae* were confirmed by polymerase chain reaction (PCR) using the 16s rRNA gene. Antibiotic susceptibility testing (AST) was done on these isolates, and the PCR was used to evaluate the common ESBL-encoding genes and carbapenem resistance genes. Genotyping was performed using multi-locus sequencing typing (MLST). Results: The overall prevalence of *K. pneumoniae* in Southwestern Nigeria was 30.5%. The AST revealed high resistance rates to tetracyclines (67.2%), oxacillin (61.7%), ampicillin (60.2%), ciprofloxacin (58.6%), chloramphenicol (56.3%), and lowest resistance to meropenem (43.0%). All isolates were susceptible to polymyxin B. The most prevalent ESBL gene was the TEM gene (47.7%), followed by CTX-M (43.8%), SHV (39.8%), OXA (27.3%), CTX-M-15 (19.5%), CTX-M-2 (11.1%), and CTX-M-9 (10.9%). Among the carbapenemase genes studied, the VIM gene (43.0%) was most detected, followed by OXA-48 (28.9%), IMP (22.7%), NDM (17.2%), KPC (13.3%), CMY (11.7%), and FOX (9.4%). GIM and SPM genes were not detected. MLST identified six different sequence types (STs) in this study. The most dominant ST was ST307 (50%, 5/10), while ST258, ST11, ST147, ST15, and ST321 had (10%, 1/10) each. Conclusion: High antimicrobial resistance in *K. pneumoniae* is a clear and present danger for managing infections in Nigeria. Additionally, the dominance of a successful international ST307 clone highlights the importance of ensuring that genomic surveillance remains a priority in the hospital environment in Nigeria.

## 1. Introduction

*Klebsiella pneumoniae* is one of the most important pathogenic bacteria in healthcare. It is a gram-negative, bacilli, nonmotile, and causative agent of many infectious diseases, such as pneumonia, sepsis, burns, wound infections, pyogenic liver abscesses, and urinary tract infections [1]. *K. pneumoniae* affects mainly patients who have predisposing debilitating backgrounds [2]. In Nigeria, *K. pneumoniae* is among the most common etiological agents of lower respiratory tract infections [3]. This bacterium has been reported to be the second most common cause of urinary tract infections [2], with an increasing rate of drug resistance to many commonly used antibiotics [4]. The emergence of carbapenem-resistant *K. pneumoniae* (CRKP) strains has become a critical challenge for public health worldwide due to their capacity to disseminate rapidly in the hospital environment [5]. The rapid spread of carbapenem-resistant *K. pneumoniae* (CRKP), listed by the WHO as a critical priority pathogen, has become a global threat to human health due to high morbidity and mortality [6].

*K. pneumoniae* utilizes different resistance mechanisms to counteract the effects of antibiotics, such as the production of destructive enzymes, target alteration, efflux pumps, and porin loss [2]. Therefore, hospital-associated infections with multidrug-resistant (MDR) strains of *K. pneumoniae* occur with high morbidity and mortality [7]. The emergence of extended-spectrum beta-lactamases (ESBL)-producing organisms was considered to be from the dissemination of clones of some epidemic strains along with the horizontal transmission of resistance gene-carrying plasmids among bacteria [8]. The development and selection of multiple drug-resistant bacteria, such as ESBL producers, have also been attributed to the rise in the use of second and third-generation cephalosporins to treat *K. pneumonia* infections [9].

Carbapenemases are enzymes that are capable of hydrolyzing the newer carbapenem antibiotics used in the treatment of MDRbacterial infections [7]. Among these, *Klebsiella pneumoniae* carbapenemase (KPC), metallo-β-lactamases (VIM, IMP, NDM), and OXA-48 types of enzymes are the most common. Mobile genetic elements, including plasmids, transposons, and integrons, are involved in disseminating related encoding genes [10]. ESBL and carbapenemase-producing organisms often acquire resistance to non-β-lactam antibiotics, including aminoglycosides and fluoroquinolones, resulting in multi-drug resistant properties.

In Nigeria, the existence of these resistance profiles has been established; however, little work has been done on the molecular identification and characterization of ESBLs and carbapenemase genes [11]. A study carried out in two tertiary hospitals in Northwest Nigeria showed that 58% of their *K. pneumoniae* and *E.coli* isolates were ESBL producers, while resistance to imipenem and meropenem was observed in 36.6% and40.3% of the isolates, respectively [12].

Factors known to promote the spread of ESBLs and carbapenemase-producing isolates include irrational use of antibiotics both in the hospital and community, suboptimal infection prevention and control practices, prolonged hospitalization, use of invasive devices (e.g., central venous lines, urinary catheters, and endotracheal tubes), stay in nursing homes, and presence of immunosuppressive conditions [13]. 

The main purpose of this study was to evaluate the antimicrobial resistance patterns and molecular mechanisms of *ESBLs* and carbapenem resistance among clinical isolates of *K. pneumoniae* from hospitalized patients in tertiary care hospitals in Southwestern Nigeria.

## 2. Materials and Methods

### 2.1. Study Site and Sample Collection

A total number of 420 clinical specimens that included urine, blood, sputum, wound swabs, high vaginal swabs (HVS), pus, stool, tracheal aspirate, and semen of patients that were diagnosed with various diseases were collected from hospitals in six states of Southwestern Nigeria. These included the Ladoke Akintola University of Technology Teaching Hospital, Osogbo, Osun State; the Obafemi Awolowo University Teaching Hospitals Complex, Ile—Ife, Osun State; the Lagos State University Teaching Hospital, Lagos State; the Federal Medical Centre Abeokuta, Ogun State; the University College Hospital, Ibadan Oyo State; the Federal Medical Centre Ido Ekiti, Ekiti State; and the Federal Medical Centre Owo, Ondo State between February 2018 and July 2019 and then transported to the medical microbiology and parasitology laboratory, the Ladoke Akintola University of Technology, Ogbomoso for microbiological and molecular analysis. Demographic and clinical information about the source of each clinical specimen were included in the data collection.

### 2.2. Isolation and Identification of Bacteria

Samples were cultured by inoculating into the blood and MacConkey agar and incubated at 37 °C for 18–24 h. Growth on blood agar and MacConkey (Oxoid Ltd., Basingstoke, Hampshire, UK) agar was identified by cultural characteristics, morphological appearance, and biochemical tests and confirmed by Microbact GNB 12E (Oxoid Ltd., Basingstoke, Hampshire, UK). All *K. pneumonia* isolates were further confirmed by polymerase chain reaction (PCR) using the 16s rRNA gene.

### 2.3. Antibiotic Susceptibility Testing

The antibiotic susceptibility testing was performed by the Kirby—Bauer Disc Diffusion and broth microdilution methods as modified by the Clinical and Laboratory Standards Institute [14]. The following antibiotic disks (Oxoid Ltd., Basingstoke, Hampshire, UK) were used: chloramphenicol (30 μg), ampicillin (10 μg), cefoxitin (30 μg), ceftriaxone (30 μg), cefuroxime (30 μg), cephalexin (30 μg), cefotaxime (30 μg), ceftazidime (30 μg), levofloxacin (1 μg), imipenem (10 μg), meropenem (10 μg), and aztreonam (30 μg), tetracycline (30 μg), gentamicin (30 μg), ciprofloxacin (5 μg), cefepime (30 μg), amikacin (30 μg), ofloxacin (5 μg), amoxicillin/clavulanic acid (30 μg), oxacillin (5 μg), and polymyxin B (300 units).

All plates were incubated at 37 °C for 24 h. The diameters of inhibition zones were measured to the nearest millimeter using a ruler. Control strain *K. pneumoniae* ATCC 700603was used in the testing to validate the results of disc diffusion.

### 2.4. Detection of Antimicrobial Resistance Determinants

DNA molecules were extracted by boiling method [15] and used to prepare the PCR reaction mixture. All isolates were analyzed for the presence of β-lactamase genes, including ESBL genes (*bla_TEM_*, *bla_SHV_*, *bla_OXA_*, *bla_CTX-M_*, *bla_CTX-M-2_*, *bla_CTX-M-9_*, *bla_CTX-M-15_*) and carbapenemases genes (*bla_FOX_*, *bla_CMY_*, *bla_KPC_*, *bla_IMP_*, *bla_VIM_*, *bla_GIM_*, *bla_SPM_*, *bla_NDM-1_* and *bla_OXA-48_*) (Table 1). At the completion of the amplification, PCR products were resolved inl.2% agarose gel stained with 0.5 μL of ethidium bromide. The DNA bands were visualized and photographed using a gel bio-imaging system (UVP Imaging System, Upland, CA, USA). The type-specific PCR products were recognized clearly by their distinct band sizes.

### 2.5. Genetic Diversity Assessment by Multi-locus Sequence Typing (MLST)

Ten isolates were randomly selected for multi-locus sequence typing (MLST). Primers, PCR reaction conditions, and detailed methodology were in accordance with those previously described by [31]. Determination of allele profiles and sequence types (STs) was conducted by comparing the obtained sequences to the documented data at Klebsiella Pasteur MLST database (https://bigsdb.web.pasteur.fr/Klebsiella/Klebsiella.html, (accessed on 25 October 2022). Table 2 shows the PCR Primers nucleotides, annealing temperatures, and product sizes.

### 2.6. Statistical Analysis

Statistical analysis was performed using the Statistical Package for Social Sciences software (SPSS version 24), and statistical significance was set at *p* < 0.05. Data were presented as frequencies and percentages.

## 3. Results

### 3.1. Distribution of Socio-Demographic Data of Selected Variables and Number of Klebsiella pneumoniae Positive Isolates

Out of the 420 samples collected, 128 (30.5%) were positive for *K. pneumoniae*. The overall prevalence of *K. pneumoniae* in Southwestern Nigeria was 30.5%. Of the *K. pneumoniae-*positive samples, Lagos state had the highest prevalence of *K.pneumoniae*, 32/70 (45.7%), followed by Oyo state, 25/70 (35.7%), while the lowest prevalence was seen in samples from Ekiti state,16/70 (22.9%). The difference in these isolation rates was statistically significant (*p* = 0.027). The highest recovery rate of *Klebsiella pneumoniae* was from tracheal aspirate specimens (42.9%) though the highest number was seen in urine specimens (40). The differences in the proportion of recovery of *K. pneumoniae* from the various sample types were not significant (*p* = 0.540). Although there were no significant differences (*p* = 0.441) in the recovery rate of *K. pneumoniae* from wards and clinics, the highest recovery rate was from the intensive care unit, where almost half of their samples (43.7%) yielded *K. pneumoniae* (Table 3).

### 3.2. Antibiotic Resistance Patterns of K. pneumoniae Isolates

The highest levels of antibiotic resistance were displayed against tetracycline (67.2%), oxacillin (61.7%), ampicillin (60.2%), ciprofloxacin (58.6%), and chloramphenicol (56.3%), while drugs with the least antibiotic resistance (below 50%) were imipenem (48.4%), cefepime (44.5%), and meropenem (43.0%). All isolates were susceptible to polymyxin B (Table 4).

### 3.3. The Distribution of the ESBL Genes Produced by the Multidrug-Resistant K. pneumoniae Isolates

**Figure 1:**
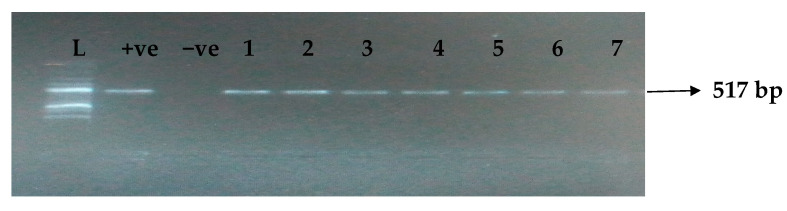
Electrophoresis gel picture of the *bla_TEM_* gene. L = 100 bp ladder, +ve = *TEM* positive control, −ve = *TEM* negative control, and 1–7 = sample representatives of *bla_TEM_*-positive isolates.

**Figure 2:**
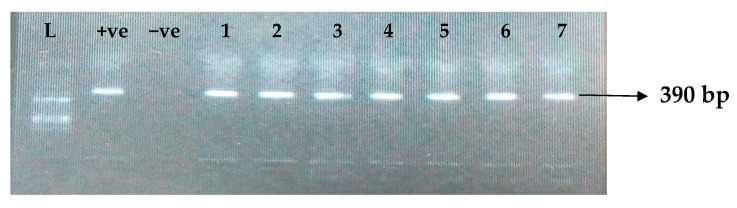
Electrophoresis gel picture of the *bla_SHV_* gene. L = 100 bp ladder, +ve = *SHV* positive control, −ve = *SHV* negative control, and 1–7 = sample representatives of *bla_SHV_*-positive isolates.

**Figure 3:**
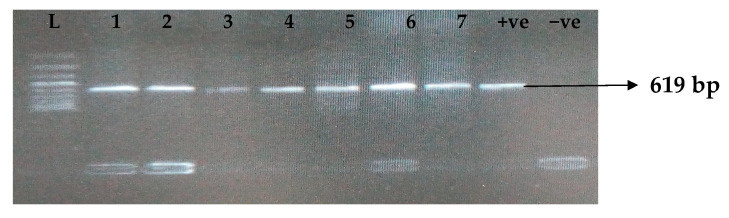
Electrophoresis gel picture of the *bla_OXA_* gene. L = 100 bp ladder, +ve = *OXA* positive control, −ve = *OXA* negative control, and 1–7 = sample representatives of *bla_OX__A_*-positive isolates.

**Figure 4:**
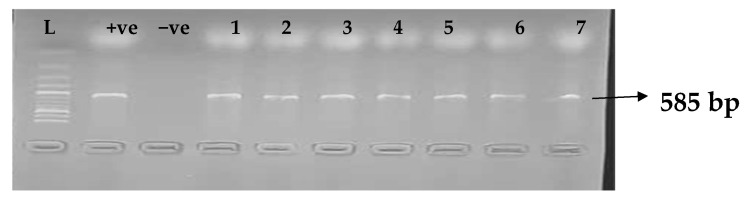
Electrophoresis gel picture of the *bla_CTX-M_* gene. L = 100 bp ladder, +ve = *CTX-M* positive control, −ve = *CTX-M* negative control, and 1–7 = sample representatives of *bla_CTX-M_*-positive isolates.

Table 5 depicts the prevalence of ESBL-associated genes in clinical isolates of *K. pneumoniae*. The TEM gene (47.7%) was recovered most, followed by CTX-M (43.8%), SHV (39.8%), OXA (27.3%), CTX-M-15 (19.5%), CTX-M-2 (11.1%), and CTX-M-9 (10.9%). The gel electrophoresis profiles of *bla_TEM_, bla_SHV_, bla_OXA,_ and bla_CTXM_* genes are presented in Figure 1, Figure 2, Figure 3 and Figure 4, respectively.

### 3.4. Distribution of Carbapenemase Genes among K. pneumoniae Isolates

**Figure 5:**
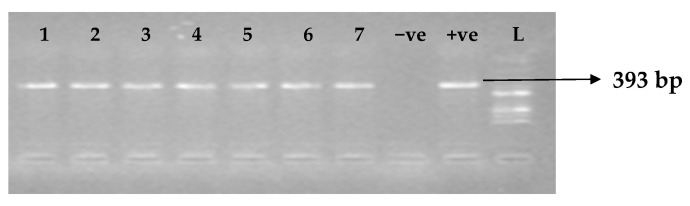
Electrophoresis gel picture of the *bla_VIM_* gene. L = 100 bp ladder, +ve = *VIM* positive control (*K. pneumoniae* ATCC 13883 strain), −ve = *VIM* negative control, and 1–7 = sample representatives of *bla_VIM_*-positive isolates.

**Figure 6:**
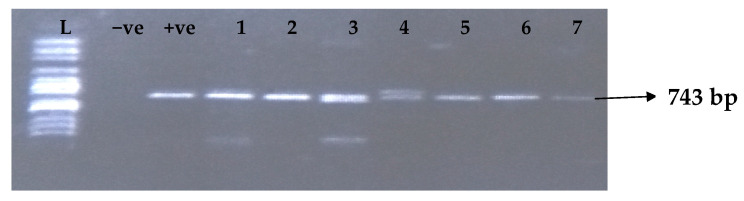
Electrophoresis gel picture of the *bla_OXA-48_*gene.L = 100 bp ladder, +ve = *OXA-*48positive control, −ve = *OXA-48* negative control, and 1–7 = sample representatives of *bla_OXA-48_*-positive isolates.

**Figure 7:**
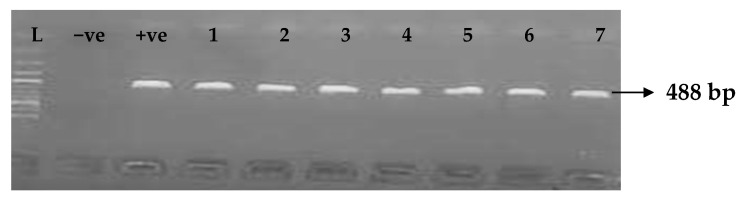
Electrophoresis gel picture of the *bla_IMP_* gene. L = 100 bp ladder, +ve = *IMP* positive control, −ve = *IMP* negative control, and 1–7 = sample representatives of *bla_IMP_*-positive isolates.

**Figure 8:**
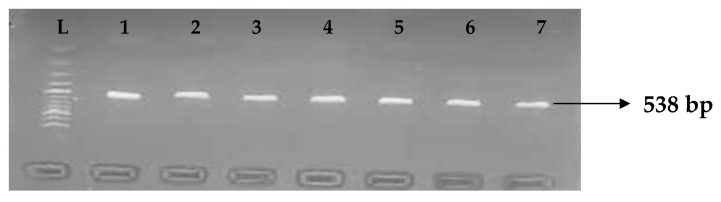
Electrophoresis gel picture of the *bla_KPC_* gene. L = 100 bp ladder and1–7 = sample representatives of *bla_IMP_*-positive isolates.

**Figure 9:**
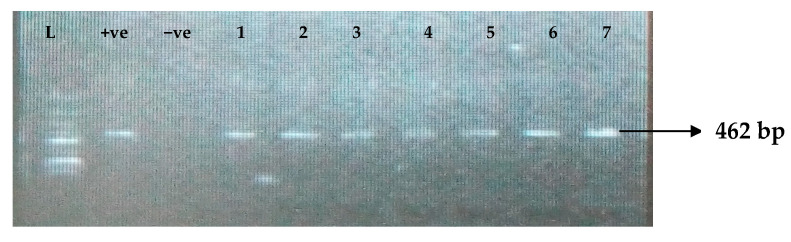
Electrophoresis gel picture of the *bla_CMY_* gene. L = 100 bp ladder, +ve = *CMY* positive control, −ve = *CMY* negative control, and 1–7 = sample representatives of *bla_CMY_*-positive isolates.

**Figure 10:**
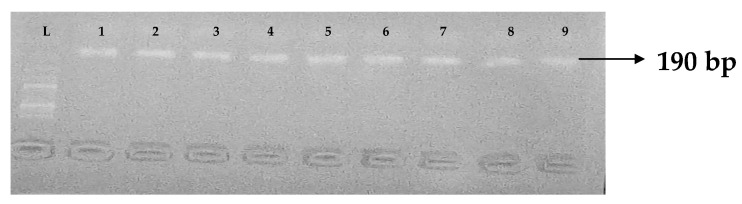
Electrophoresis gel picture of the *bla_FOX_* gene. L = 100 bp ladder and 1–9 = sample representatives of *bla_FOX_*-positive isolates.

Carbapenemases are a group of beta-lactamases that are able to breakdown the active core of carbapenems antibiotics. Table 6 depicts the distribution of different carbapenemase-associated genes from the clinical isolates of *K. pneumoniae*. The VIM gene (43.0%) was most detected among the clinical isolates, followed by OXA-48 (28.9%), IMP (22.7%), NDM (17.2%), KPC (13.3%), CMY (11.7%), and FOX (9.4%). GIM and SPM were not detected. The gel electrophoresis profiles of *bla_VIM_*, *bla_OXA__-48_*, *bla_IM__P_*, *bla_KPC_*, *bla_CM__Y,_* and *bla_FOX_* genes are presented in Figure 5, Figure 6, Figure 7, Figure 8, Figure 9 and Figure 10, respectively. 

Table 7 shows relationship between phenotypic and genotypic genes of *K. pneumoniae*. From the table, carbapenem phenotype showed a significant relationship with KPC, IMP, and VIM genes.

### 3.5. Genetic Diversity Assessment by MLST

Table 8 shows the results of MLST conducted on 10 *K. pneumoniae* to determine the extent of genotypic diversity among the *K. pneumoniae* isolates. Results from the table revealed that six different sequence types (STs) were identified in this study. The most dominant ST was ST307 (50%, 5/10), while ST258, ST11, ST147, ST15, and ST321 had (10%, 1/10) each.

## 4. Discussion

*K. pneumoniae* has been reported as one of the main pathogens causing nosocomial and community-acquired infections in humans over a long period of time. Due to antimicrobial resistance, treatment of *K. pneumonia* infections has become complicated and difficult to treat [32]. The 30.5% prevalence of *K. pneumoniae* in this study is similar to the 34% reportedin Lagos state and also in the southwest [33]. Hence, it can be inferred that *K. pneumoniae* is associated with clinical infections in Southwest Nigeria. Similar findings have been reported in other parts of the country, such as 30.0% recorded in Kano State [34]; it is, however, higher than 12.8%reported in Kaduna State [35]. The high prevalence rate of *K. pneumoniae* observed in this study could be explained by the fact that all isolates investigated in this study were sourced from hospitalized patients, which may underscore the lack of proper infection control practices [36], showing that *K. pneumoniae* is a common nosocomial pathogen [37]. There was an association between isolated *K. pneumoniae* and the selected six states showing that isolation of *K. pneumoniae* depends on the hospital or its site.

The result of this study revealed that *K. pneumoniae* infection was seen more in females than males. The higher occurrence of these isolates among females might result from the higher prevalence of urogenital *K. pneumoniae* isolates in our study. The major proportion of samples used in this study were urine samples. Hence, the highest number of *K. pneumoniae* was observed in the urine sample, and this is in agreement with the finding of [38].

A high rate of antimicrobial resistance was observed in our study, as more than half of the isolates were resistant to most antibiotics tested. The 48.4% and 43.0% resistance rates of clinical *K. pneumoniae* isolates to imipenem and meropenem in this study are similar to the observation in Ebonyi, Nigeria, where [39] reported 41.1% for imipenem and 43.3% for meropenem. However, our observed resistance rate to imipenem is higher than 24% in Oyo State [40] and 19.05% in Kaduna State [41], also in Nigeria. According to previous studies, imipenem and meropenem have shown good activity against Enterobacteriaceae [42]; therefore, the findings of this study show that there has been a steady increase in resistance to these antibiotics over the years. This may be as a result of their increasing use among the populace.

In the current study, we observed that ESBL-KP isolated from different clinical samples harbor multiple ESBL genes (*blaCTX-M*, *blaTEM*, *bla_OXA,_* and *blaSHV*), which is similar to other studies, including a study from India [43].The most prevalent ESBL gene in this study was *bla_TEM,_* with a 47.7% prevalence rate comparable to 49.3% reported in India [44] and 52% reported in Southwestern Nigeria [45]. However, this prevalence is in sharp contrast to the 100% reported in Port Harcourt [46] and 14.28% in Sokoto State [35]. The 39.8% prevalence of the *bla_SHV_* gene among clinical *K. pneumonia* isolates reported in this study is comparable to the 35% reported in Pakistan [47]. This current prevalence is lower than 58.33% reported in Port Harcourt [46] and 48% in Southwestern Nigeria [45]. In addition, the 27.3% prevalence rate of the *bla_OXA_* gene among clinical *K. pneumoniae* isolates is lower than the 65% reported in Pretoria [48] and 41.67% in Port Harcourt [46].

It has been proven that the *bla_CTX-M-15_* among humans has increased outstandingly over time in most countries. The 43.8% prevalence of the *bla_CTX-M_* gene among our clinical *K. pneumoniae* isolates is slightly higher than the 41.67% reported in Port Harcourt two years earlier [46] and also higher than 35.71% in Sokoto State [35] and the 32%in Southwestern Nigeria four years ago [45]. The 19.5% prevalence of the *bla_CTX-M-15_* among clinical *K. pneumoniae* isolates is comparable to the 12.5% prevalence in China [49] and 14.54% in Iran [50]. However, [47] reported a 46% prevalence of the *CTX-M-15* gene in Pakistan. Our study adds to the body of evidence that the *CTX-M-15* remains the most important *CTX-M* enzyme in *K. pneumoniae* as a result of its large diffusion and relation to infections in humans. Similarly, this particular genotype is widely disseminated in Africa [51].

Moreover, 13.3% of the clinical *K. pneumoniae* isolates possessed the *CTX-M-2* gene, which is lower than the 45.7% reported in Argentina [52]. Additionally, the 10.9% prevalence of the *bla_CTX-M-9_* among clinical *K. pneumoniae* isolates is comparable to the 9.69% in China [53]. However, [54] reported a 40% prevalence of the *CTX-M-9* gene in Saudi Arabia, which is significantly greater than the study’s prevalence rate. The coexistence of ESBL genes in these isolates may have also contributed to the observed high rate of antimicrobial drug resistance [55]. These data have clinical applications for selecting empiric antibiotic therapy when infections caused by ESBL-producing *K. pneumoniae* are suspected [55].

The present work corroborates the findings of [56], who reported that *bla_VIM_* was frequently involved in causing carbapenem resistance in humans. Similarly, [56] also reported that *bla_VIM_* (69.2%) was the predominant gene in hospitalized patients in Egypt. The 43.0% prevalence rate of the *bla_VIM_* gene among carbapenemase-producing *K. pneumoniae* in this study is higher than the 33.3% reported in Iran [57] but lower than the 84.62% in Egypt [58]. However, in contrast to our findings, no clinical isolate of *K. pneumonia* harbored the *VIM* in a Brazilian study [7].

We report a lower prevalence of the *bla_KPC_* gene among carbapenemase-producing *K. pneumonia* compared to the *blaVIM* gene. Similar low proportions have been reported by [59] in Jos, Plateau state, and even a much lower prevalence of 2.7%in Port Harcourt, Nigeria [60]. Our findings were contrary to the zero prevalence reported in South Africa, which could be because these were mainly surveillance studies conducted among asymptomatic persons [61]. It could also be attributed to the restricted use of antibiotics in those countries as opposed to Nigeria, where antibiotics are easily available over-the-counter. We did not find any *bla_SPM_* or *bla_GIM_* genes in *K. pneumoniae* isolates. This is in agreement with other studies reporting that these genes are limited to distinct geographical regions such as Germany and Brazil [62].

Molecular studies showed the prevalence of AmpC genes were 11.7% and 9.4% for *bla_CMY_* and *bla_FOX_*, respectively. Similarly, in the Zorgani study in Tripoli, the majority of AmpC-positive isolates (66.6%) were found to carry the CMY-encoding gene [63]. The possible reason for this prevalence may be due to excessive usage of extended-spectrum cephalosporin in the treatment of gram-negative infections [64].

In this study, MLST showed that the *K. pneumoniae* strains belonged to six different sequence types (STs), revealing clonal diversity. ST307 was the most concentrated, accounting for 5 (50%). This finding is in accordance with the report of [65], who reported ST307 as the most prevalent ST in Southwestern Nigeria. All five isolates having ST307 were obtained from the urine. These isolates were also found to have similar genotypes regarding ESBLs (CTX-M-15) and carbapenemase (KPC). Several countries, such as Italy, Korea, the USA, Mexico, and China, have reported carbapenem-resistant *K. pneumoniae* ST307 with ESBL production [66]. The ST307 identified in our study were present in five tertiary hospitals in Southwestern Nigeria, indicating the role of immigration in the transmission of these successful international clones from diverse geographical settings.

It is of note in Africa that the problem of carbapenem-resistant Enterobacteriaceae (CRE) is becoming increasing on a daily basis, especially in Nigeria, where the usage of carbapenem is on the increase in our clinical settings, as expressed from our data. It is of note that other factors contributed to this aggravated increase by other factors such as the issue of poor diagnostic tools in our tertiary health care settings, poor sanitation and dirty environment linked to the high rate of infections, sub-optimal disease surveillance, and incessant over-the-counter abuse and usage of antibiotics. It is of note that the burden and the problem of CRE in Africa are underreported [11,67].

## 5. Conclusions

A total number of 128 non-duplicate *K. pneumoniae* were isolated and characterized from hospitalized patients in Southwestern Nigeria. The high MDR *K. pneumoniae* observed in this study is worrisome and calls for action. Factors such as the frequent use of carbapenems and cephalosporins, as well as the lack of antibiotic therapy policies and guidelines in most healthcare facilities in the country, should be addressed as these could be responsible for the observed high level of resistance. This study also demonstrates that although there is considerable diversity among the *K. pneumoniae* in Nigerian hospitals, a high proportion of the isolates belonged to one clonal group; therefore, molecular epidemiological surveillance and control can effectively reduce the occurrence and spread of drug-resistant bacterial infections in hospitalized patients.

## Figures and Tables

**Table 1:** Primers used to amplify genes encoding beta-lactamase in *K. pneumoniae.*

Primer	Sequence 5^1^–3^1^	Annealing Temperature	Product Size (bp)	Reference
16 s rRNA F 16 s rRNA R	ATTTGAAGAGGTTGCAAACGAT TTCACTCTGAAGTTTTCTTGTGTTC	57 °C	130	[16]
TEM-H F TEM-H R	CCCCGAAGAACGTTTTC ATCAGCAATAAACCAGC	52 °C	517	[17]
SHV-1 F SHV-1 R	AGGATTGACTGCCTTTTTG ATTTGCTGATTTCGCTCG	57 °C	393	[17]
OXA F OXA R	ATATCTCTACTGTTGCATCTCC AAACCCTTCAAACCATCC	57 °C	619	[17]
CTX-M F CTX-M R	CGATGTGCAGTACCAGTAA TTAGTGACCAGAACAGCGG	57 °C	585	[18]
CTX-M-2 F CTX-M-2 R	ATGATGACTCAGAGCATTCG GAAACCGTGGGTTACGATTT	60 °C	1400	[19]
CTX-M-9 F CTX-M-9 R	GTGACAAAGAGAGTGCAACGG ATGATTCTCGCCGCTGAAGCC	60 °C	857	[20]
CTX-M-15 F CTX-M-15 R	CCATGGTTAAAAAATCACTGCG TGGGTRAARTARGTSACCAGAAYSAGCGG	60 °C	805	[21]
KPC F KPC R	CATTCAAGGGCTTTCTTGCTGC ACGACGGCATAGTCATTTGC	55 °C	538	[22]
NDM F NDM R	CACCTCATGTTTGAATTCGCC CTCTGTCACATCGAAATCGC	58 °C	984	[23]
VIM2004A VIM2004B	GTTTGGTCGCATATCGCAAC AATGCGCAGCACCAGGATAG	54 °C	390	[24]
IMP F IMP R	CATGGTTTGGTGGTTCTTGT ATAATTTGGCGGACTTTGGC	55 °C	488	[25]
SPM F SPM R	CCTACAATCTAACGGCGACC TCGCCGTGTCCAGGTATAAC	55 °C	650	[26]
GIM F GIM R	AGAACCTTGACCGAACGCAG ACTCATGACTCCTCACGAGG	55 °C	599	[27]
CMY F CMY R	TGGCCAGAACTGACAGGCAAA TTTCTCCTGAACGTGGCTGG	47 °C	462	[28]
FOXMF FOXMR	AACATGGGGTATCAGGGAGATG CAAAGCGCGTAACCGGATTGG	54 °C	190	[29]
OXA 48F OXA 48R	TTGGTGGCATCGATTATCGG GAGCACTTCTTTTGTGATGGC	55 °C	743	[30]

Source: Inqaba Biotec, Pretoria, South Africa.

**Table 2:** Primer sequences, annealing temperatures, and PCR product sizes for MLST.

Primer	Sequence 5^1^–3^1^	Annealing Temperature	Product Size (bp)
rpoBF rpoBR	GGCGAAATGGCWGAGAACCA GAGTCTTCGAAGTTGTAACC	50 °C	501
gapA F gapA R	TGAAATATGACTCCACTCACGG CTTCAGAAGCGGCTTTGATGGCTT	60 °C	450
mdh F mdh R	CCCAACTCGCTTCAGGTTCAG CCGTTTTTCCCCAGCAGCAG	50 °C	477
pgi F pgi R	GAGAAAAACCTGCCTGTACTGCTGGC CGCGCCACGCTTTATAGCGGTTAAT	50 °C	432
phoE F phoE R	ACCTACCGCAACACCGACTTCTTCGG TGATCAGAACTGGTAGGTGAT	50 °C	420
infB F infB R	CTCGCTGCTGGACTATATTCG CGCTTTCAGCTCAAGAACTTC	50 °C	318
tonB F tonB R	CTTTATACCTCGGTACATCAGGTT ATTCGCCGGCTGRGCRGAGAG	45 °C	414

**Table 3:** Distribution of socio-demographic data of selected variables and number of *Klebsiella pneumoniae*-positive isolates.

Variable		Number (%)	Positive No(%)	Pearson Chi-Square	df	*p*-Value
Location	Ekiti Lagos Ogun Ondo Osun Oyo	70 (16.7) 70 (16.7) 70 (16.7) 70 (16.7) 70 (16.7) 70 (16.7)	16 (22.9) 32 (45.7) 17 (24.3) 20 (28.6) 18 (25.7) 25 (35.7)	12.631	5	0.027
	Total	420	128 (30.5)			
Age	≤10 years	19 (4.5)	7 (36.8)	0.634	3	0.889
	11–25 years	63 (15)	19 (30.2)			
	26–50 years	209 (49.8)	61 (29.2)			
	51 years and above	129 (30.7)	41 (31.8)			
Sex	Female	220 (52.4)	69 (31.4)	0.172	1	0.679
	Male	200 (47.6)	59 (29.5)			
Sample	Blood	34 (8.0)	7 (20.6)	6.968	8	0.540
	High Vaginal Swab	62 (14.8)	20 (32.3)			
	Pus	65 (15.5)	25 (38.5)			
	Semen	7 (1.7)	1 (14.3)			
	Sputum	45 (10.7)	12 (26.7)			
	Stool	14 (3.3)	4 (28.6)			
	Tracheal Aspirate	7 (1.7)	3 (42.9)			
	Urine Wound Swab	144 (34.3) 42 (10)	40 (27.8) 16 (38.1)			
Ward	Accident and emergency	10 (2.38)	2 (20)			
	Intensive Care Unit	16 (3.81)	7 (43.7)			
	Geriatrics	25 (5.95)	10 (40)			
	Medical	160 (38.1)	41 (25.6)			
	Obstetrics and Gynaecology	96 (22.9)	32 (33.3)			
	Pediatrics	13 (3.1)	4 (30.8)			
	Surgical	100 (23.8)	32 (32)			

**Table 4:** Antibiotic resistance patterns of *K. pneumoniae* isolates.

S/N	Antibiotics	Resistant Number/Percentage
1	Levofloxacin	66 (51.6)
2	Cefoxitin	69 (53.9)
3	ceftazidime	68 (53.1)
4	tetracycline	86 (67.2)
5	aztreonam	70 (54.7)
6	gentamicin	69 (53.9)
7	Cefepime	57 (44.5)
8	Imipenem	62 (48.4)
9	Amikacin	70 (54.7)
10	meropenem	55 (43.0)
11	Ofloxacin	69 (53.9)
12	cephalexin	69 (53.9)
13	Amoxycillin/Clavulanic acid	68 (53.1)
14	ciprofloxacin	75 (58.6)
15	Cefuroxime	74 (57.8)
16	ampicillin	77 (60.2)
17	oxacillin	79 (61.7)
18	Cefotaxime	66 (51.6)
19	chloramphenicol	72 (56.3)
20	Ceftriaxone	69 (53.9)
21	Polymyxin B	0 (0.0)

**Table 5:** Distribution of the ESBL genes produced by the multidrug-resistant *K. pneumonia* isolates.

ESBL Genes	Frequency	Percent
TEM	61	47.7
SHV	51	39.8
OXA	35	27.3
CTX-M	56	43.8
CTX-M-2	15	11.1
CTX-M-9	14	10.9
CTX-M-15	25	19.5

**Table 6:** Distribution of carbapenemase genes among *K. pneumoniae* isolates.

	Frequency	Percent
VIM	55	43.0
OXA-48	37	28.9
IMP	29	22.7
NDM	22	17.2
KPC	17	13.3
CMY	15	11.7
FOX	12	9.4
SPM	0	0.0
GIM	0	0.0

**Table 7:** Analysis of carbapenem phenotypes and carbapenem genes.

Antibiotics	Number/% Resistant (Phenotype)	KPC Gene (17)		IMP Gene (29)		VIM Gene (55)	
		Number (%)	*p* Value	Number/%	*p* Value	Number/%	*p* Value
Levofloxacin	66 (51.6)	2 (11.8)	0.505	16 (55.2)	0.062	29 (52.7)	0.480
Cefoxitin	69 (53.9)	4 (23.5)	0.447	11 (37.9)	0.510	32 (58.2)	0.254
Ceftazidime	68 (53.1)	1 (5.9)	0.274	14 (48.3)	0.107	29 (52.7)	0.540
Tetracycline	86 (67.2)	6 (35.2)	0.381	17 (58.6)	0.403	37 (67.3)	0.570
Aztreonam	70 (54.7)	5 (29.4)	0.325	12 (41.4)	0.406	33 (60.0)	0.193
Gentamicin	69 (53.9)	4 (23.5)	0.447	14 (48.3)	0.130	31 (56.4)	0.380
Cefepime	57 (44.5)	2 (11.8)	0.315	8 (27.6)	0.395	27 (49.1)	0.235
Imipenem	62 (48.4)	5 (29.4)	0.041 *	16 (55.2)	0.000 *	30 (54.5)	0.036 *
Amikacin	70 (54.7)	4 (23.5)	0.486	8 (27.6)	0.181	32 (58.2)	0.305
Meropenem	55 (43.0)	8 (47.1)	0.001 *	16 (55.2)	0.000 *	35 (63.6)	0.000 *
Ofloxacin	69 (53.9)	7 (41.2)	0.081	12 (41.4)	0.361	33 (60.0)	0.154
Cephalexin	69 (53.9)	3 (17.6)	0.553	13 (44.8)	0.230	32 (58.2)	0.254
Amoxycillin/Clavulanic acid	68 (53.1)	1 (5.9)	0.274	6 (20.7)	0.076	28 (50.9)	0.398
Ciprofloxacin	75 (58.6)	7 (41.2)	0.209	14 (48.3)	0.342	36 (65.4)	0.118
Cefuroxime	74 (57.8)	3 (17.6)	0.359	12 (41.4)	0.556	31 (56.4)	0.457
Ampicillin	77 (60.2)	7 (41.2)	0.270	11 (37.9)	0.271	34 (61.8)	0.441
Oxacillin	79 (61.7)	10 (58.8)	0.041	15 (51.7)	0.377	41 (74.5)	0.080
Cefotaxime	66 (51.6)	1 (5.9)	0.343	12 (41.4)	0.273	31 (56.4)	0.222
Chloramphenicol	72 (56.3)	3 (17.6)	0.436	9 (31.0)	0.221	28 (50.9)	0.190
Ceftriaxone	69 (53.9)	4 (23.5)	0.447	9 (31.0)	0.342	31 (56.4)	0.380

NOTE *: Carbapenem phenotype showed a significant relationship with KPC, IMP, and VIM.

**Table 8:** Sequence types of multidrug-resistant and hypervirulent *Klebsiella pneumoniae*.

Isolate	State	Sample	Beta-Lactamase Genes	Allelic Profile	MLST	Cloner Cluster (CC)
LK16	Lagos	Urine	*CTX-M-15*, *TEM*, *SHV*, *KPC*	3-3-1-1-1-1-4	ST11	258
LK23	Lagos	Urine	*CTX-M-15*, *VIM*, *OXA-48*, *KPC*	3-4-6-1-7-4-38	ST147	147
LK27	Lagos	HVS	*CTX-M-15*, *VIM*, *OXA-48*, *KPC*, *NDM*	4-1-2-52-1-1-7	ST307	307
OGK1032	Ogun	Pus	*CTX-M-15*, *VIM*, *KPC*, *NDM*	4-1-2-52-1-1-7	ST307	307
OYK39	Oyo	Urine	*CTX-M-15*, *VIM*, *OXA-48*, *KPC*	1-1-1-1-1-1-1	ST15	15
OYK24	Oyo	Sputum	*CTX-M-15*, *VIM*, *OXA-48*, *KPC*, *NDM*	4-1-2-52-1-1-7	ST307	307
EK55	Ekiti	Urine	*CTX-M-15*, *VIM*, *OXA-48*, *KPC*, *NDM*	4-1-2-52-1-1-7	ST307	307
ONK74	Ondo	Wound swab	*TEM*, *CTX-M-15*, *KPC*, *NDM*	3-3-1-1-1-1-79	ST258	258
OSK12	Osun	Urine	*CTX-M-15*, *KPC*, *OXA-48*, *NDM*	4-1-2-52-1-1-7	ST307	307
OSK16	Osun	Urine	*CTX-M-15*, *OXA-48*	4-16-2-1-28-3-40	ST321	321

## Data Availability

All relevant data are within the manuscript. The datasets used and/or during the current study are available from the corresponding author upon reasonable request.
